# A case for implementing an HSV1/2, VZV, and syphilis lesion panel in Manitoba, Canada

**DOI:** 10.1128/spectrum.00600-24

**Published:** 2024-06-25

**Authors:** Adam Hedley, Jared Bullard, Paul Van Caeseele, Souradet Shaw, Raymond Tsang, David C. Alexander, Kerry Dust, Derek R. Stein

**Affiliations:** 1 Cadham Provincial Laboratory, Shared Health, Winnipeg, Manitoba, Canada; 2 Department of Medical Microbiology, Rady Faculty of Health Sciences, University of Manitoba, Winnipeg, Manitoba, Canada; 3 Department of Pediatrics & Child Health, University of Manitoba, Winnipeg, Manitoba, Canada; 4 Department of Community Health Sciences, Rady Faculty of Health Sciences, University of Manitoba, Winnipeg, Manitoba, Canada; 5 Vaccine Preventable Bacterial Diseases, National Microbiology Laboratory, Public Health Agency of Canada, Winnipeg, Manitoba, Canada; Quest Diagnostics, Secaucus, New Jersey, USA

**Keywords:** syphilis, lesion assay, serology

## Abstract

Syphilis, caused by *Treponema pallidum* subsp. *pallidum* (TPA), is becoming a significant public health concern, with rising incidence in Manitoba exceeding the national average. The province has also seen a demographic shift leading to women representing 51.9% of cases in 2021, leading to the re-emergence of congenital syphilis. Given the similarities in lesion appearance between TPA and other pathogens such as herpesviruses, accurate diagnosis is crucial for effective management and prevention. In order to address the potential for missed TPA cases, we conducted a quality assurance study from June 2021 to March 2023, screening over 5,000 mucocutaneous lesion swabs for TPA, initially submitted for herpes simplex virus (HSV) and varicella zoster virus (VZV) testing. Positivity rates were 13% for HSV1, 13% for HSV2, 6.7% for VZV, and 6.6% for TPA. Turnaround times (TAT) for TPA testing, as a send-out to the reference laboratory, averaged 17.8 days. Of the TPA-positive specimens, 36% did not have a corresponding TPA PCR test ordered, and 19% did not have accompanying syphilis serology within 30 days of collection. Creation of a multiplex lesion panel identified high sensitivity and specificity for HSV1, HSV2, VZV, and TPA, with robust reproducibility across multiple runs. Incorporation of TPA into a lesion panel improved the TAT to 4 days. Our findings emphasize the need for improved testing strategies to combat the syphilis epidemic and enhance public health outcomes.

**IMPORTANCE:**

Syphilis resurgence has become a significant global public health concern. In particular, the Canadian Prairies have been struggling with high incidence since 2016, exceeding the national Canadian average. We undertook a quality assurance study that highlighted significant gaps in diagnosis of acute syphilis, which led to the development of a highly sensitive and specific multiplex lesion assay for the dual detection of herpes simplex virus type 1 (HSV-1), herpes simplex virus type 2 (HSV-2), varicella zoster virus (VZV), and syphilis.

## INTRODUCTION

*Treponema pallidum* subsp. *pallidum* (TPA), the etiological agent of syphilis, is contracted primarily through direct contact with active primary or secondary lesions, commonly occurring during sexual activity. Additionally, it can be vertically transmitted from an infected mother to her fetus during pregnancy (1). Although transmission through blood products was more prevalent in the past, improved screening and storage measures have significantly reduced its occurrence (2, 3). The disease has a progression that, if left untreated, follows through primary, secondary, latent, and sometimes tertiary stages (1). The presentation of syphilis is dependent on the stage of disease and has been nicknamed “the Great Imitator” due to its similar presentation to many diseases (4). The typical TPA lesion presents as a painless chancre at the site of inoculation during the primary stage, which can often go unnoticed, making clinical diagnosis of acute syphilis challenging. A latent period follows in which the disease is usually only detectable by serology (1). Tertiary syphilis takes years or even decades for clinical presentation with symptoms of severe cardiovascular, neurologic, skin, tissue, and bone complications (5). However, with widespread antibiotic use, unrecognized and latent infections are often inadvertently treated before tertiary syphilis develops (1).

The increased prevalence of TPA in recent years is of great public health concern (5). Over the last couple of decades, the rates of syphilis in Manitoba have exploded, increasing from 0.4 per 100,000 people in 2009 to 118 per 100,000 people in 2020, exceeding the national average of 24.6 per 100,000 (6, 7). Manitoba was also the first province to experience the demographic shift of cases from the men who have sex with men (MSM) community to heterosexual groups, which is of particular concern as women now account for 51.9% of cases in 2021, up from only 22% in 2015 (8, 9). This has also led to the re-emergence of congenital syphilis, something not seen in Manitoba since 1977, and the subsequent increasing incidence from 1 case in 2015 to 54 cases in 2021 (9, 10).

The ulcerative/vesicular lesions of herpes simplex virus (HSV1/2), varicella zoster virus (VZV), and primary syphilis can be similar in appearance, potentially resulting in the misdiagnosis of TPA infections as herpesviruses (11, 12). HSV typically causes orolabial (cold sores) or genital infections (13, 14). Traditionally, HSV1 has been transmitted through direct contact with saliva, whereas HSV2 has primarily spread through sexual contact. Primary HSV disease episodes and re-activation from latency tend to result in the “classic” presentation of painful localized vesicles progressing to ulcers (13, 15). Atypical HSV infections can also result in fissures, excoriations, vulvar erythema, gingivostomatitis, whitlow, and herpes gladitorum (16). Primary infection with the highly contagious VZV can cause a pruritic rash that, in successive waves, originates on the face or trunk and spreads to the extremities. Cutaneous VZV lesions will progress from macules to vesicular lesions before crusting over and healing; however, mucosal vesicles will rapidly rupture forming shallow ulcers (17). Due to atypical presentations associated with TPA, as well as other exanthematous pathogens like HSV1/2 or VZV, there is the potential for disease misclassification. With the increasing incidence of syphilis on the Canadian prairies, we undertook a quality assurance study to identify missed cases of primary TPA infection and evaluated the feasibility of a multiplexed lesion panel that could improve time to diagnosis and may accelerate treatment, improving public health outcomes.

## MATERIALS AND METHODS

### Study design and specimens

Mucocutaneous lesion specimens, submitted as flocked swabs in universal transport media (UTM) (cat# 220528, Becton Dickinson and Company, Franklin Lakes, NJ, USA), are routinely submitted to Cadham Provincial Laboratory for herpes virus nucleic acid testing and are stored frozen at −20°C. Between 2021 and 2023, 5,107 convenience specimens representing 4,613 patients were chosen at random for testing on an in-house TPA PCR assay (described below). This study was conducted solely under the fulfilment of ongoing quality assurance, exempt under the Canadian Tri-Council Policy Statement: Ethical Conduct for Research Involving Humans, TCSP2. All clinical specimens were de-identified and used solely with intent to evaluate the performance of our assays. Basic demographic information was collected including age, sex, and specimen site. Matching specimens with a separate TPA PCR request forwarded to the National Microbiology Laboratory (NML) were also identified. In order to account for delays in sampling and transportation, matched specimens were only considered to be associated with a given clinical investigation if ordered ±30 days from the original HSV/VZV request.

DNA extractions were performed on the KingFisher Flex instrument (Thermo Fisher Scientific, Waltham, MA, USA) with the Applied Biosystems 5x MagMAX-96 Viral Isolation Kit (cat#1836-5, Thermo Fisher Scientific, Waltham, MA, USA). Using a modified protocol and the deep-well plate format, 200 µL of sample was added to 620 µL of lysis binding solution/bead mix. Sample lysis was followed with two rounds of 300 µL of wash 1, two rounds of 300 µL of wash 2, and a 110 µL elution step. Extracted DNA was stored at 4°C.

### PCR assays

After routine testing for HSV1-HSV2-VZV, the extracted DNA was screened for TPA using real-time PCR to detect the *Tp47* gene. This was carried out using TaqMan Fast Virus 1-Step Master Mix (cat# 4444432, Thermo Fisher Scientific, Waltham, MA, USA) at a final volume of 20 µL, including 5 µL of template DNA, and previously published primers and probe (18): 5′-CAACACGGTCCGCTACGACTA-3′, 5′-TGCCATAACTCGCCATCAGA-3′, and 5′-HEX-CGGTGATGACGCGAGCTACACCA-BHQ1-3′. The PCR was run on a CFX-96 Real-Time System (Bio-Rad Laboratories, Hercules, CA, USA) with the following conditions: one cycle of 50°C for 5 minutes, one cycle of 95°C for 20 seconds, followed by 40 cycles of 95°C for 5 seconds and 61.4°C for 30 seconds with plate read. CFX Maestro software (v 2.3) was used to analyze the PCR data.

### Lesion panel PCR development and validation

The primers and probes targeting the TPA *Tp47* gene were incorporated into the previously validated HSV/VZV PCR to create a multiplex lesion panel PCR run on the Bio-Rad CFX-96 thermal cycler (19, 20). This manual multiplex lesion panel was then adapted to the fully automated Hologic Panther Fusion instrument (Hologic Canada, Mississauga, ON, Canada). The multiplex assay was optimized using Hologic’s myAccess software (version 2.1.2.1). The Panther Fusion extraction and PCR parameters were customized as follows: sample aspiration height: low. Extraction type: viral, RNA/DNA; FCR-S/FER-S; 360 µL extraction. Elution buffer volume was set to 50 µL, and the template volume to 5 µL. The thermal cycling profile was as follows: one cycle at 95°C for 2 minutes, followed by 45 cycles of 95°C for 7 seconds and 58°C for 30 seconds. The curve correction analysis parameters were left at the default settings. The Ct thresholds were set to 100 cycles for channels 1 (FAM), 2 (HEX), 3 (ROX), and 4 (Q670). The threshold for channel 5 (Q705) was set to 75 cycles. Most crosstalk settings were left at the default value of 0%, but to avoid signal bleed-through between FAM/HEX and ROX/Q670, the “emit channel 1/receive channel 2” and “emit channel 3/receive channel 4” settings were adjusted to 1% and 2%, respectively. The slope settings and the background fluorescence settings were left at the default settings. Five hundred microliters of UTM was transferred into an Aptima Specimen Lysis tube prior to testing (Cat# PRD-04339, Hologic Canada, Mississauga, ON, Canada).

### Multiplex lesion panel

Archived samples submitted to the lab for HSV, VZV, or TPA PCR were used for clinical sensitivity and specificity experiments, whereas the previously validated PCR methods served as the comparator assays. The primer and probe sequences, as well as concentrations used in this multiplex PCR, are listed in Table S1 and S2.The analytical sensitivity of the lesion panel PCR was determined by testing dilutions of quantified commercial standards for each target: NATrol Herpes Simplex Virus Type 1 (cat# NATHSV1-0005), NATrol Herpes Simplex Virus Type 2 (cat# NATHSV2-0005), NATrol Varicella-Zoster Virus (cat# NATVZV-0005) (ZeptoMetrix, Buffalo, NY, USA); Synthetic *Treponema pallidum* DNA (ATCC number BAA-2642SD). Probit analyses, as described by Westgard and Westgard, were used to calculate the limit of detection for each target (21). To verify analytical specificity, various viruses and bacteria were tested. To measure the inter- and intra-assay reproducibility, positives for each of the targets were tested over three separate runs or a single run, and the coefficient of variation was calculated.

## RESULTS

### Evaluation of syphilis screening in Manitoba

From June 2021 to March 2023, we tested 5,107 mucocutaneous specimens, initially submitted for HSV and VZV investigation, for TPA using an in-house PCR assay. Sixty percent of the specimens collected were from women, whereas 40% were from men. Patients ranged in age from newborn to 98 years old with the majority of specimens in the 20- to 59-year age group (61% of all positives). The overall positivity rates for HSV1, HSV2, VZV, and TPA were 13%, 13%, 6.7%, and 6.6%, respectively, among all specimens (Table 1). HSV1-positive specimens were observed in all age groups with the highest being individuals aged 20–39 years (44% of all HSV1-positive specimens). HSV2 was not detected among the youngest group but was found in all other ages with the 20- to 39-year-olds having the highest HSV2 positivity (58% of all HSV2-positive specimens). VZV positivity was lowest in the 0–9 age group, whereas both the 20–39 and 60+ groups had the highest rates. TPA positivity was low in children 0–9 years of age, whereas considerably elevated in age groups of sexual maturity, with 60% of TPA-positive specimens coming from 20- to 39-year-olds. Considering the sampled population, the positivity of HSV1, HSV2, and VZV was similar among the various geographic health regions of Manitoba. Notably, 70% of the TPA-positive specimens were from Winnipeg (38%) and the Northern health region (32%). HSV1 positivity was highest in oral, female genital, and other cutaneous sites (34%, 31%, and 23%, respectively) but relatively low in male genital (7%) and rectal/perineal sites (4.5%). In contrast, HSV2 was observed in only 0.6% of positive oral sites. The majority of VZV positivity (77%) was observed in cutaneous specimens. TPA positivity was much higher in male and female genital specimens (36% and 40% of positive specimens, respectively) compared to rectal/perineal specimens (6.9%).

**TABLE 1:** The demographics of specimens screened (sex, age, RHA,^*a*^ sample type) by our HSV-VZV LDT^*b*^ and the TPA screening assay

Characteristics		HSV-1	HSV-2	VZV	TPA (LDT)
	Overall	Detected	Detected	Detected	Detected
	*N* = 5,107 (100%)	*N* = 670 (13%)	*N* = 657 (13%)	*N* = 343 (6.7%)	*N* = 334 (6.5%)
Sex					
Female	3,042 (60%)	458 (68%)	446 (68%)	212 (62%)	188 (56%)
Male	2,052 (40%)	209 (31%)	211 (32%)	130 (38%)	145 (43%)
Unknown	13 (0.3%)	3 (0.4%)	0 (0%)	1 (0.3%)	1 (0.3%)
Age group					
0–9	625 (12%)	71 (11%)	0 (0%)	11 (3.2%)	16 (4.8%)
10–19	480 (9.4%)	96 (14%)	47 (7.2%)	19 (5.5%)	53 (16%)
20–39	2,097 (41%)	292 (44%)	384 (58%)	116 (34%)	199 (60%)
40–59	1,034 (20%)	127 (19%)	163 (25%)	80 (23%)	59 (18%)
60+	871 (17%)	84 (13%)	63 (9.6%)	117 (34%)	7 (2.1%)
Regional health authority					
Winnipeg	2,854 (56%)	372 (56%)	368 (56%)	207 (60%)	127 (38%)
Prairie Mountain	449 (8.8%)	73 (11%)	57 (8.7%)	30 (8.7%)	34 (10%)
Interlake East	377 (7.4%)	48 (7.2%)	38 (5.8%)	28 (8.2%)	33 (9.9%)
Northern	714 (14%)	64 (9.6%)	128 (19%)	35 (10%)	107 (32%)
Southern	465 (9.1%)	83 (12%)	42 (6.4%)	33 (9.6%)	16 (4.8%)
Unknown	248 (4.9%)	30 (4.5%)	24 (3.7%)	10 (2.9%)	17 (5.1%)
Specimen type					
Female genital	1,287 (25%)	210 (31%)	321 (49%)	46 (13%)	132 (40%)
Male genital	708 (14%)	47 (7.0%)	186 (28%)	19 (5.5%)	119 (36%)
Rectal/perineal	192 (3.8%)	30 (4.5%)	40 (6.1%)	8 (5.5%)	23 (6.9%)
Oral	709 (14%)	227 (34%)	4 (0.6%)	7 (2.0%)	34 (10%)
Cutaneous	2,211 (43%)	156 (23%)	106 (16%)	263 (77%)	26 (7.8%)

^*a*^ RHA; regional health authority

^*b*^ LDT; laboratory developed test

**Fig 1:**
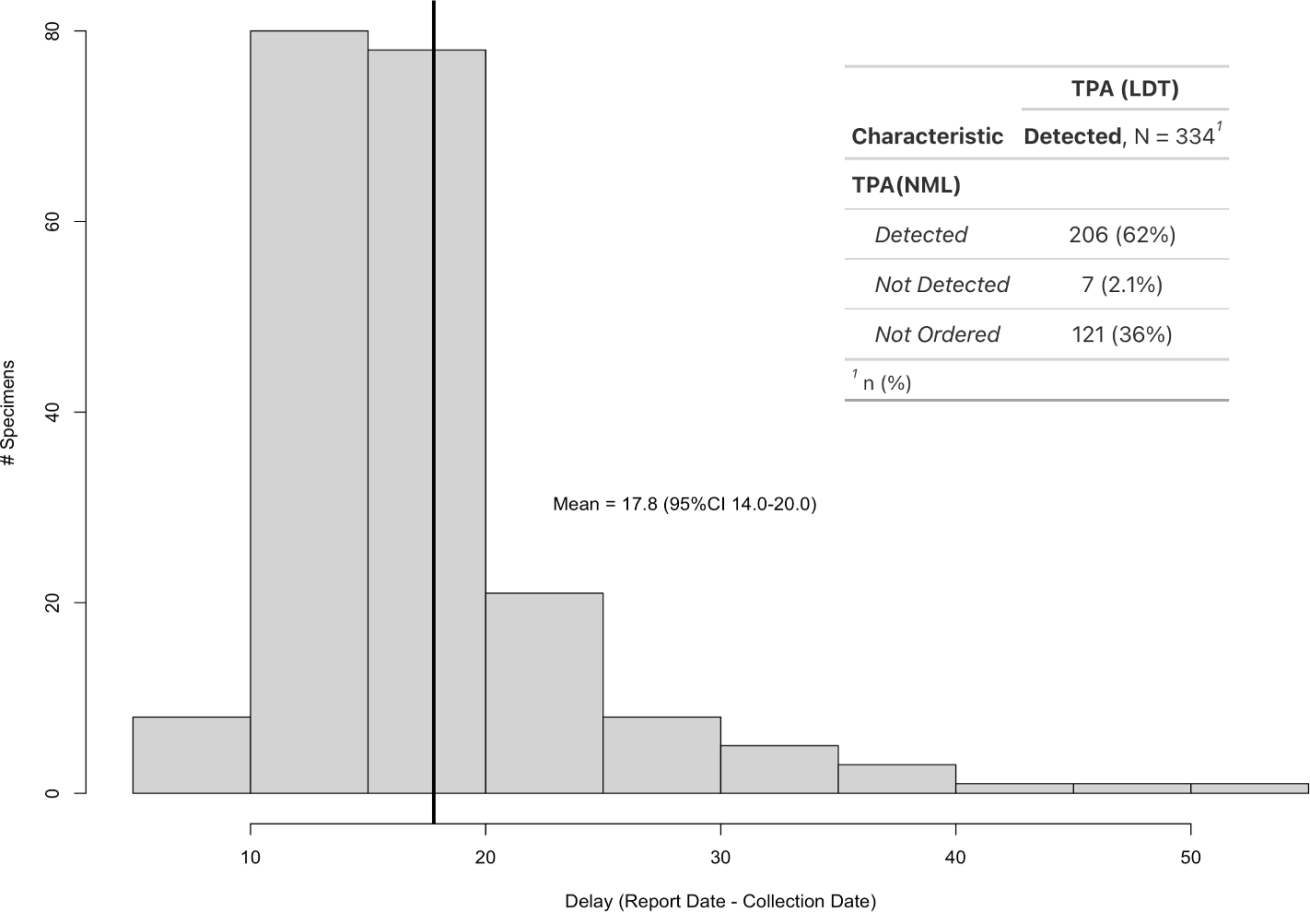
The time from collection to result for samples forwarded to the reference lab for TPA detection. RHA; regional health authority. LDT; laboratory developed test.

Of the 334 TPA-positive specimens detected by our in-house screening assay, only 62% had a TPA PCR test ordered by a health care practitioner within ±30 days of the HSV/VZV specimen submission (Fig. 1). Conversely, 121 specimens (36%) that tested positive by our in-house assay did not have a corresponding TPA PCR request ordered by a health practitioner. In addition, seven specimens (2.1%) with a corresponding TPA PCR test were negative by the reference laboratory. Upon further investigation, five out of the seven specimens were confirmed as positive by serological follow-up. Interestingly, a single infant tested positive by the in-house TPA assay on three separate specimens (ear, mouth, and rectal) despite not having received a separate request for TPA PCR from the ordering practitioner. Further investigation showed there was concurrent positive syphilis serology done. Of the 206 specimens that tested positive for TPA by the NML, the mean turnaround time (TAT) was 17.8 days (95% confidence interval [CI] 14.0–20.0). In some instances, the TAT was as much as 52 days.

Serology is the standard of care for syphilis screening and diagnosis; thus, the absence of a TPA PCR request does not necessarily rule out clinically suspected cases of syphilis. Of the 121 TPA-positive samples that did not have an NML TPA PCR ordered, 65 did not have an accompanying syphilis serology request within a 30-day period (data not shown). Of the 56 specimens that did have an accompanying syphilis serology request, six specimens were classified as serology negative despite having detectable bacteria by PCR. Relying on the serology to detect early cases would have led to an average potential reporting delay of 7.3 days (95% CI 5.0–7.0) with respect to acute syphilis infections when comparing the HSV/VZV specimen collection date to the accompanying serology report date.

### Creation of a multiplex lesion panel

In order to identify potential cases of acute syphilis with improved reporting delay, we developed a multiplex lesion panel on the random access Hologic Panther Fusion. Archived clinical samples (*n* = 146), originally submitted for HSV/VZV or TPA PCR testing, were used to determine the sensitivity and specificity of the new panel. The previously validated diagnostic methods served as the gold standard comparators. Sensitivity was 100% for HSV1 and VZV, and 96% for HSV2 and TPA. Clinical specificity was 99% for HSV1, and 100% for HSV2, VZV, and TPA (Table 2).

**TABLE 2:** Performance characteristics of the multiplex lesion panel with archived samples

Target	HSV1	HSV2	VZV	TPA
Performance characteristics				
No. of true positives	30	27	27	45
No. of false positives	1	0	0	0
No. of true negatives	115	118	119	99
No. of false negatives	0	1	0	2
Sensitivity	100%	96%	100%	96%
Specificity	99%	100%	100%	100%
Kappa agreement	0.98	0.98	1	0.97
95% CI	0.94 to 1.00	0.93 to 1.00	1.00 to 1.00	0.93 to 1.00
Standard error	0.01	0.04	0	0.02

Dilutions of commercially quantified HSV1, HSV2, VZV, and TPA samples were made to determine the limit of detection and gauge the sensitivity of the lesion panel. Probit calculations determined the lesion panel PCR limit of detection to be 171 cp/mL for HSV1, 187 cp/mL for HSV2, 629 cp/mL for VZV, and 193 cp/mL for TPA. Cross-reactivity was assessed using archived samples positive for other viruses and bacteria. No false amplification was observed for samples known to be positive for human papillomavirus types 16 and 18, human enterovirus, adenovirus, measles, mumps, cytomegalovirus, monkeypox, *Chlamydia trachomatis*, or *Neisseria gonorrhoeae*. Inter- and intra-assay precision was also determined, with the coefficient of variation for all targets under 5% (Tables S3 and S4).

## DISCUSSION

This study stemmed from a concern about the rising incidence of TPA infections in our province and the myriad of clinical presentations manifested. Its reputation as “the Great Imitator” suggests that some syphilis cases could be erroneously attributed to other pathogens, leading to misdiagnosis. To address this concern, we screened over 5,000 mucocutaneous lesion specimens illuminating several critical aspects of TPA infections while identifying gaps and quality improvement measures that should be applied to Manitoba’s syphilis testing practices. Historically, with low TPA prevalence, the current process of utilizing the expertise of the off-site reference lab was sufficient. The turnaround time for the majority of specimens was within 10–20 days (average 17.8 days). However, considering the ongoing outbreak in Manitoba, improving test turnaround time is key to ensuring timely treatment and prevention of transmission.

Of the 5,107 specimens, 2,572 had a matching syphilis serology request within the time period studied (June 2021–March 2023). This indicates that only 50% of individuals presenting with mucocutaneous lesions are being screened for syphilis. Of particular concern is that acute TPA cases may be missed due to failure to request molecular testing when suspect lesions are present. We identified 121 TPA-positive specimens without a request for TPA PCR, of which only 50 (41.3 %) had follow-up confirmatory positive serology requested. This indicates that 71 acute infections may have gone undiagnosed and identifies potential gaps with respect to delayed treatment. Furthermore, a limitation of our study is the absence of treatment data, making it challenging to fully quantify the true number of missed cases due to aggressive clinical treatment. We also identified six TPA PCR-positive specimens that were serologically negative. It is possible these patients may have not yet seroconverted, and accurate diagnosis of acute TPA infection was missed. Serology results can also be complicated by previous infections, whereas PCR results will indicate active infection. The results from our screening assay demonstrate that there are missed opportunities in diagnosing active TPA infections. Historically, separate swabs from the same lesion are required for herpesvirus and TPA testing at separate laboratories. Paired samples where the first swab captures more material than the second may lead to suboptimal sensitivity.

In order to improve acute TPA diagnosis, sample workflow, and turnaround time, we developed a multiplex lesion panel, which was highly sensitive, specific, and reproducible, allowing for comprehensive orolabial and genital ulcer disease testing from a single specimen on a single platform. Implementation of this assay realized an improvement in TAT from an average of 17 days down to 4 days. The lesion panel also allows for acute cases to be classified as lab-confirmed rather than probable based on serology, which would otherwise require further follow-up, and a potential delay in treatment. This lesion assay has some potential limitations. Because the primers and probes used to detect TPA are not specific for *T. pallidum* subspecies *pallidum*, non-venereal treponematoses (e.g., *T. pallidum* subspecies *endemicum* and *T. pallidum* subspecies *pertenue*) could also be detected. However, unlike syphilis, yaws and bejel are rare in Manitoba, and they can be distinguished from syphilis with supplementary testing (22).

Manitoba is in the midst of a syphilis epidemic with rates currently exceeding the national average of 24.6 per 100,000 (7). Here, we reviewed Manitoba’s syphilis screening and testing procedures, and identified areas for improvement. We have developed a convenient and sensitive molecular assay to simultaneously detect syphilis, herpes simplex 1 and 2, and varicella zoster from lesions. We propose that implementation of this lesion panel will improve syphilis detection in Manitoba and across Canada.

## SUPPLEMENTAL MATERIAL

Supplemental tables spectrum.00600-24-s0001.docx Table S1–S4.

ASM does not own the copyrights to Supplemental Material that may be linked to, or accessed through, an article. The authors have granted ASM a non-exclusive, world-wide license to publish the Supplemental Material files. Please contact the corresponding author directly for reuse.
