# Supplementary material for: A case for implementing an HSV1/2, VZV, and syphilis lesion panel in Manitoba, Canada
Source: Microbiol Spectr. 2024 Jun 25;12(8):e00600-24. doi: 10.1128/spectrum.00600-24 (PMC11302493; doi:10.1128/spectrum.00600-24)
Supplement: Supplemental tables — Table S1–S4. [file spectrum.00600-24-s0001.docx]

**Supplementary Data**

**Table S1.** Oligonucleotide sequences for the Multiplex Lesion Panel

| **Name** | **Oligonucleotide Sequence** | **Target** | **Reference** |
| --- | --- | --- | --- |
| HSV1 | 5’-GCAGTTTACGTACAACCACATACAGC-3’ | HSV1 gB | (Namvar *et al.*) |
| HSV2 | 5’-TGCAGTTTACGTATAACCACATACAGC-3’ | HSV2 gB | (Namvar *et al.*) |
| HSV3 | 5’-AGCTTGCGGGCCTCGTT-3’ | HSV1 /2 gB | (Namvar *et al.*) |
| HSV4 | 5’-(FAM/ZEN)CGGCCCAACATATCGTTGACATGGC-3’ | HSV1 gB | (Namvar *et al.*) |
| HSV5 | 5’-(HEX/ZEN)- CGCCCCAGCATGTCGTTCACGT-3’ | HSV2 gB | (Namvar *et al.*) |
| Tpal4 | 5’-CAACACGGTCCGCTACGACTA-3’ | Tpal tpp47 | (Gayet-Ageron *et al.*) |
| Tpal5 | 5’-TGCCATAACTCGCCATCAGA-3’ | Tpal tpp47 | (Gayet-Ageron *et al.*) |
| Tpal6 | 5’-(TXRed-XN)CGGTGATGACGCGAGCTACACCA-BHQ2-3’ | Tpal tpp47 | (Gayet-Ageron *et al.*) |
| VZV5 | 5’-CGGCATGGCCCGTCTAT-3’ | VZV DNA pol | (Weidmann *et al.*) |
| VZV6 | 5’-TCGCGTGCTGCGGC-3’ | VZV DNA pol | (Weidmann *et al.*) |
| VZV7 | 5’-(CY5/TAO)ATTCAGCAATGGAAACACACGACGCC-3’ | VZV DNA pol | (Weidmann *et al.*) |
| BG1 | 5’-TGGATGAAGTTGGTGGTGAG-3’ | Betaglobin | CPL- unpublished |
| BG2 | 5’-CCCAGTTTCTATTGGTCTCCTT-3’ | Betaglobin | CPL- unpublished |
| BG3 | 5’-(Q705)CCTGGGCAGGTTGGTATCAAGGTT-BHQ2-3’ | Betaglobin | CPL- unpublished |

**Table S2.** Concentrations for the multiplex lesion panel

| **Component** | **Stock Concentration (mM)** | **PPR Concentration** | |
| --- | --- | --- | --- |
| **Buffers** |  |  |  |
| MgCl_2_ | 1000 | 3.75 | mM |
| KCl | 2000 | 125 | mM |
| 1X TE buffer: Tris-HCl | 10 | 11.4 | mM |
| 1X TE buffer: 0.1M EDTA | 0.1 | 0.11 | mM |
| **Oligonucleotide Primers** |  |  |  |
| HSV1 | 0.1 | 0.50 | µM |
| HSV2 | 0.1 | 0.50 | µM |
| HSV3 | 0.1 | 0.50 | µM |
| HSV4 | 0.1 | 0.25 | µM |
| HSV5 | 0.1 | 0.25 | µM |
| VZV5 | 0.1 | 0.63 | µM |
| VZV6 | 0.1 | 0.63 | µM |
| VZV7 | 0.1 | 0.63 | µM |
| Tpal4 | 0.1 | 0.63 | µM |
| Tpal5 | 0.1 | 0.63 | µM |
| Tpal6 | 0.1 | 0.31 | µM |
| BG1 | 0.1 | 0.13 | µM |
| BG2 | 0.1 | 0.13 | µM |
| BG3 | 0.1 | 0.25 | µM |

**Table S3.** Inter-assay reproducibility for each target tested over three separate runs. A stronger and a weaker sample were tested for each pathogen.

| **Target** | | **HSV1** | **HSV2** | **VZV** | **TPA** |
| --- | --- | --- | --- | --- | --- |
| **Sample 1** | |  |  |  |  |
|  | Average Ct value | 21.00 | 28.63 | 18.07 | 28.00 |
|  | Standard Deviation | 0.53 | 1.00 | 0.12 | 0.35 |
|  | Coefficient of Variation (%) | 2.50 | 3.50 | 0.60 | 1.20 |
| **Sample 2** | |  |  |  |  |
|  | Average Ct value | 30.87 | 33.27 | 36.60 | 33.83 |
|  | Standard Deviation | 0.64 | 0.23 | 0.28 | 0.46 |
|  | Coefficient of Variation | 2.10 | 0.70 | 0.80 | 1.40 |

**Table S4.** Intra-assay reproducibility for each target testing in triplicate on a single run. A stronger and a weaker sample were tested for each pathogen.

| **Target** | | **HSV1** | **HSV2** | **VZV** | **TPA** |
| --- | --- | --- | --- | --- | --- |
| **Sample 1** | |  |  |  |  |
|  | Average Ct value | 23.63 | 22.97 | 21.30 | 28.47 |
|  | Standard Deviation | 0.06 | 0.99 | 0.36 | 0.12 |
|  | Coefficient of Variation (%) | 0.20 | 4.30 | 1.70 | 0.40 |
| **Sample 2** | |  |  |  |  |
|  | Average Ct value | 28.17 | 28.87 | 29.50 | 33.83 |
|  | Standard Deviation | 0.12 | 0.38 | 0.17 | 0.46 |
|  | Coefficient of Variation | 0.40 | 1.30 | 0.60 | 1.40 |
